# The Chinese version of the Quality of Life in Childhood Epilepsy Questionnaire-16-C (QOLCE-16-C): translation, validity, and reliability

**DOI:** 10.1186/s12955-022-01960-8

**Published:** 2022-03-28

**Authors:** Ping Tang, Qunfeng Lu, Yinghui Wu, Lin Wang, Wenjuan Tang, Yan Jiang, Liling Yang, Jianlin Ji, Xiaomin Sun, Jingmin Sun, Jie Yang

**Affiliations:** 1 Department of Nursing, Shanghai Children’s Hospital, Shanghai Jiao Tong University, Shanghai, 200062 People’s Republic of China; 2 Department of Neurology, Shanghai Children’s Hospital, Shanghai Jiao Tong University, Shanghai, 200062 People’s Republic of China; 3 Department of Outpatient, Shanghai Children’s Hospital, Shanghai Jiao Tong University, Shanghai, 200062 People’s Republic of China; 4 School of Nursing, Shanghai Jiao Tong University, Shanghai, 200025 People’s Republic of China

**Keywords:** Epilepsy, Pediatrics, Health related quality of life, Translation, Factor analysis, Reliability and validity

## Abstract

**Background:**

Epilepsy is one of the most common chronic neurological diseases that adversely impact the quality of life of patients and their families. The “Quality of Life of Childhood Epilepsy Questionnaire” (hereinafter referred to as “QOLCE-16”) is a 16-item measure that was designed to assess health-related quality of life (HRQOL) among children with epilepsy. The purpose of the study was to translate and evaluate the psychometric properties of the QOLCE-16.

**Methods:**

The 10 steps of Principles of Good Practices for translation and cultural adaptation of measures were adopted to translate the QOLCE-16 into Chinese. After that, item analysis, floor effect and ceiling effect, internal consistency, test–retest reliabilities, content validity and construct validity were conducted to test its applicability in children with epilepsy in China. A total of 435 native Chinese-speaking parents with children who had epilepsy from one children’s hospital were invited to take part in the study, including a cognitive interview sample of 5 and a validation sample of 430.

**Results:**

A total of 414 objects were enrolled in our study for psychometric testing. The results of the item analysis revealed QOLCE-16-C to have good discrimination, the floor effect and ceiling effect were 0.2% and 1.0% respectively, and each item was significantly related to the total scale (*P* < 0.001). The Cronbach’s α value was 0.938 and the test–retest reliability was 0.724. For validity, results showed that the QOLCE-16-C had good content validity. Exploratory factor analysis indicated it was reasonable that the QOLCE-16-C consists of four dimensions after rotation. Confirmatory factor analysis demonstrated good construct validity (χ^2^/df = 1.698, GFI = 0.913, CFI = 0.974, RMSEA = 0.058).

**Conclusion:**

The Chinese version of QOLCE-16-C appears to be a culturally appropriate, valid and reliable tool to assess the health-related quality of life of children with epilepsy in China.

## Plain English summary

Epilepsy is one of the most common chronic neurological diseases. Due to its high prevalence and serious adverse effects, epilepsy has become a major public health concern. Health related quality of life (HRQOL) is a multidimensional concept, used to obtain a wider range of treatment and recovery information for patients in clinical practice. As such, the ability to precisely measure HRQOL becomes critical for children living with epilepsy. To date, several inventories have been established to evaluate the HRQOL of children with epilepsy, among them, QOLCE-16 not only has adequate measurement characteristics, but also possesses a small number of items, which making it suitable for the busy clinical work in China. Despite this, there is no Chinese version of the scale in China at present. In this study, we have translated the QOLCE-16 from English into Chinese. Followed by this translation is an assessment of the psychometric properties of the Chinese version of the Quality of Life in Childhood Epilepsy Questionnaire-16-C (QOLCE-16-C). This study indicates that the Chinese version of QOLCE-16-C appears to be a culturally appropriate, valid and reliable tool to assess the health related quality of life of children with epilepsy in China.

## Background

Epilepsy is one of the most common chronic neurological diseases, which is characterized by abnormal brain discharge accompanied with unpredictable and recurrent seizures [1]. Studies have shown that there are roughly 9.84 million people with epilepsy in mainland China, which most of these being pediatric patients [2]. Children with epilepsy are experiencing issues not only associated with neurological deficits and functional limitations, but are also going through profound psychiatric and psychosocial influences compared to patients with other chronic diseases [3–5]. Moreover, children with epilepsy are limited in social activities, face future driving restrictions, and discrimination with social stigma, which may lead to a decrease in self-esteem and increased negative emotions (e.g., depression and social isolation), all of which could pose obstacles for improving their quality of life [6]. High prevalence and serious adverse effects have made epilepsy as one of the major public health concerns [7].

Considering epilepsy’s chronic nature and significant negative effects on multiple facts of children’s lives, traditional treatments for seizure control no longer meet the current focus of long-term care for epilepsy. Thus, reducing seizure frequency and improving the quality of life should be regarded as the primary goals of antiepileptic treatments [8]. Health related quality of life (HRQOL) is a multidimensional concept, which is used to obtain a wider range of treatment and recovery information of patients in clinical practice [9–11]. Measurement of HRQOL in children with epilepsy helps to accurately reflect their physical, emotional, and psychosocial functions [12]. Hence, the ability to precisely measure HRQOL becomes critical for children living with epilepsy.

To date, several inventories have been established to evaluate the HRQOL of children with epilepsy, such as the Quality of Life in Epilepsy for Adolescents (QOLIE-AD-48) [13], the Quality of Life in Childhood Epilepsy Questionnaire (QOLCE-55) [14], and the Health-Related Quality of Life Measure for Children with Epilepsy (CHEQOL-25) [15]. However, the relatively lengthy items of these tools have cost reporters large amounts of time and patience to complete. Additionally, management burdens have been given to medical staff, which may limit its practical use in busy clinical research settings, especially China.

In order to minimalize the burden of respondents, Goodwin and other scholars recently developed a 16-item-version measure (QOLCE-16) from the QOLCE-55 that allows for the capturing of HRQOL while maintaining the strong properties of the original tool based on the item response theory methods [8, 16]. The reliability and sensitivity of the QOLCE-16 was tested across age, sex, and time, which shows this short version is both time-saving and appropriate as measure for clinic [8].

Although QOLCE-16 is an excellent and practical inventory for China’s busy clinical and scientific environment, its use in China is currently blank due to the absence of a Chinese version. The purpose of this study was to first translate the QOLCE-16 from English into Chinese, followed by an assessment of the psychometric properties of the Chinese version of the Quality of Life in Childhood Epilepsy Questionnaire-16-C (QOLCE-16-C). Expectations for the Chinese version of the QOLCE-16-C were the following: (1) appropriate cultural equality; (2) high internal consistence; (3) good test–retest reliability; (4) strong structural validity.

## Methods

This study consists of two phases: (1) translation of the QOLCE-16 to Chinese, followed by a cross-cultural adaptation among the parents with children with epilepsy; (2) testing of the psychometric properties of the QOLCE-16-C, including reliability and validity assessments.

### Phase 1: translation and cross-cultural adaptation procedures

**Fig. 1:**
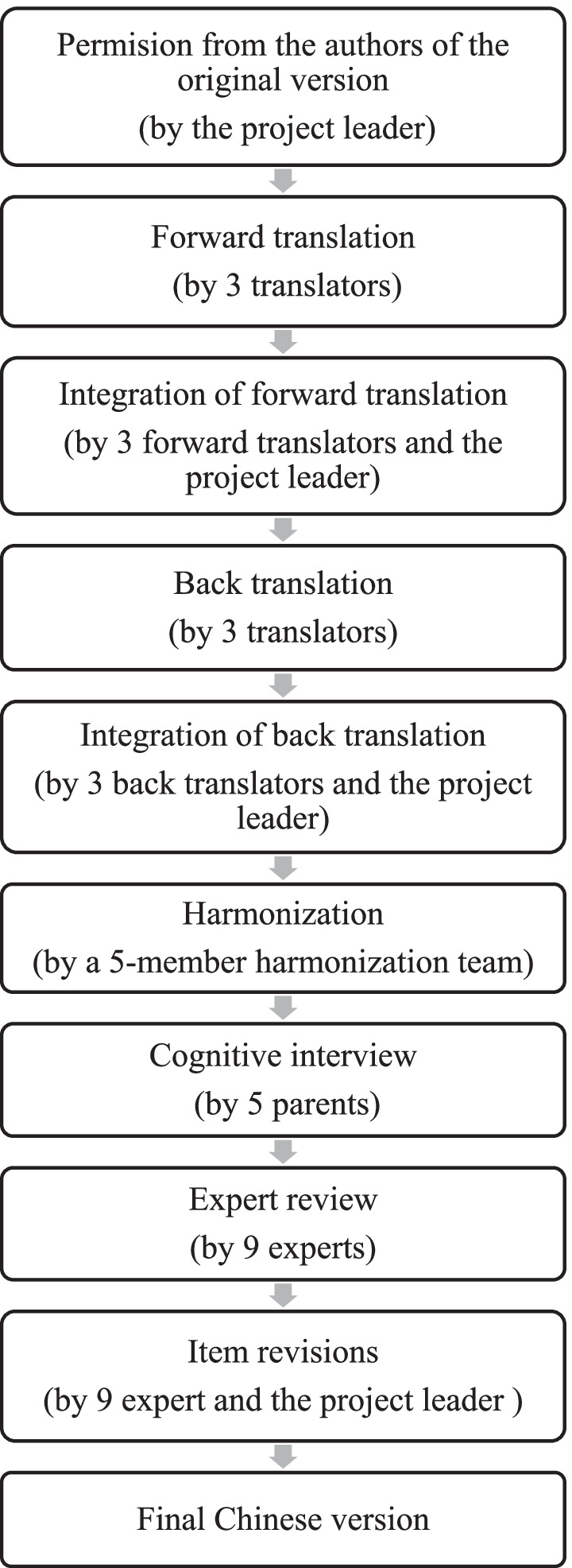
Translation of the QOLCE-16

The goal of phase 1 was to produce a semantically equivalent, grammatically fluent, culturally compatible, and readily comprehensible QOLCE-16-C. Due to the lack of gold translation standards [17], we adopted the 10 steps of Principles of Good Practices for Translation and Cultural Adaptation of measures established by the International Society for Pharmacoeconomics and Outcomes Research (ISPOR) [18], which has commonly been used in large international researches, including the Patient-reported outcome measurement information system (PROMIS) [19], the World Health Organization Quality of Life (WHOQOL) [20], the Neuro-quality of life (Neuro-QoL) [21], and others [22, 23]. Figure 1 shows the specific translation steps.

#### The forward and backward translation

After receiving permission for translating from the development group of QOLCE-16 (Professor Kathy N. Speechley) through E-mails, the forward translation team (1 pediatric neurology nurse, 2 graduate students) translated the instrument independently. All three of them were bilingual with experience living in English-speaking countries for at least one year. The forward translated team that translated the English version were then reconciled into one through discussion among the three translators as well as the leader of the project. After that, another group of three bilingual translators (1 child neurologist, 1 child health doctor, 1 English-speaking translator) who were blind to the study objects and original QOLCE-16 conducted the back-translation. After discussion, an integrated and back-translated English version was obtained.

#### Harmonization

A 5-member harmonization team (1 linguistic specialist, 2 medical school teachers with experience in scale translation research, 2 pediatric nursing doctoral students) conducted an evaluation and comparison between the original scale, forward version and back-translated version to produce an agreement on a draft version.

#### Cross-cultural adaption

To evaluate the accuracy of the draft version with the original version, the cognitive interviewing technique was used in 5 parents whose child had epilepsy. Prior to the cognitive interview, an interview outline was developed and evaluated by experts to ensure its rationality. According to the interview outline, parents were asked about their understanding of the whole scale and the expression of specific items. A 10-point scale was used to measure how well parents understood the items. A rating of “1” meant that the item was difficult to answer, while a rating of “10” meant that the item was easy to answer. General probing and paraphrasing techniques were used for some of the items, which consist of asking respondents to explain the questions, requesting them to define meanings of words used in the questions, explaining their responses and identifying areas of the questionnaire that pose difficulty in understanding, interpretation or completion [24, 25].

#### Expert review

The Delphi Method was used during this procedure [26]. 9 experts (1 child neurologist, 1 child neurosurgeon, 1 child health doctor, 1 child rehabilitation doctor, 1 pediatrician in public health, 2 professors of nursing, 1 head nurse of neurosurgery, and 1 head nurse of neurology) were invited to make comparison among the above original scale, forward version, back-translated version, and cognitive interview integrated version through E-mail, and to form the final integrated version, the QOLCE-16-C.

### Phase 2: QOLCE-16-C psychometric testing procedure

#### Study settings and subjects

##### Settings

Parents with children who had epilepsy from Shanghai Children’s hospital were invited to take part in the study. A convenience sampling method was adopted to recruit the eligible participants in the Neurology department’s inpatient and outpatient clinics.

##### Acceptance and row standard

The inclusion criteria were children who were (1) aged 4–18 years, (2) with a diagnosis of epilepsy, (3) with parents able to speak and read Chinese, and (4) willing to participate in this study and have their parent’s permission. Parents who have cognitive or mental impairment and refuse to participate in the investigation were excluded.

##### Informed consent

When children and their parents came to the clinic, the researchers explained the purpose of this study to the participants and asked them if they would be willing to participate in the survey. After obtaining the written informed consent of the participants, data were aggregated through self-filled questionnaires which were filled out in the doctor’s office.

##### Sample size

It is generally considered that two independent sample sizes of 200 were deemed accepted for exploratory factor analysis (EFA) and confirmatory factor analysis (CFA) [27]. Taking into account the possibility of missing data, a final total of 430 questionnaires were distributed from June to November 2020, of which 414 were effectively returned, with an effective response rate of 96.3% (16 scales were missing data). Of these, a sample of 203 respondents was recruited to conduct the EFA, and 211 were recruited for CFA. The data collection was divided into two steps: the data for EFA was firstly collected, and then to collected data for CFA. To assess the test–retest reliability, 40 parents were randomly selected to answer the QOLCE-16-C again 2–3 weeks after their first completement. The test–retest data was collected in two ways: paper questionnaires and electronic questionnaires filled out on the WeChat platform. Finally, 32 valid retest questionnaires were recovered.

#### Instruments

##### General demographic scale

The general demographic scale was used to obtain demographic data, including information about children with epilepsy and family information. Data related to children with epilepsy included gender, age, grade of study and information related to epilepsy disease. Family data included parents' age, education background, family monthly income, payment methods for medical expenses.

##### Chinese version of the QOLCE-16

This is a short-form version of the QOLCE-55 [14] and provides an overall assessment of parent-reported HRQOL of children with epilepsy aged 4–18 years across four domains: cognitive (4 items), emotional (4 items), social (4 items), and physical functioning (4 items). The internal consistency reliability of the original QOLCE-16 was excellently high (α = 0.90) [16]. A 5-point Likert scale was used to calculate the scores for each domain and the scores for the total scale: 1 = very often, 2 = fairly often, 3 = sometimes, 4 = almost never, 5 = never [16]. The reverse code included the following items: item d of the emotional domain, and items a, b, c of the physical functioning domain. These items were then transformed as follows: response option 1 transformed to response option 5, response option 2 transformed to response option 4, response option 4 transformed to response option 2, and response option 5 transformed to response option 1. Linearly transform items from the precoded numeric values of items to a 0–100 point scale, with higher converted scores reflecting better quality of life. Responses were transformed such that: response option 1 = 0, response option 2 = 25, response option 3 = 50, response option 4 = 75, response option 5 = 100, presenting total scores take values of the unweighted mean of the four subscales from 0 (low HRQOL) to 100 (high HRQOL) [8, 14]. If more than one subscale is missing, the total score should be set to missing. It is worth noting that the scale also included the sixth option: 6 = not applicable, however this option was not incorporated into the scoring. If the answer to an item is “not applicable”, the item will be considered as missing data. The translation procedure of the Chinese version of the QOLCE-16 has been reported in phase 1.

#### Data analysis

IBM SPSS Statistics 23.0 and SPSS Amos24.0 (Armonk, NY, US) were used for analyzing the demographic characteristics and psychometric properties.

Descriptive statistics were accepted to illustrate the participants.

##### Item analysis

In item analysis, the normality of data distribution was visually inspected. Floor and ceiling effects were respectively defined as the number of participants with the highest or lowest scores on a scale. A percentage of 15% or higher indicated poor performance in the questionnaire [28]. Independent-samples for the T test were chosen to calculate the critical ratio (CR) for every item, where a value above 3 was considered statistically significant [29]. After that, the correlation coefficient between each item and the total scale scores was checked, with a value greater than 0.4 considered to have a good correlation between the two [29].

##### Reliability

Reliability was tested by internal consistency and test–retest reliability. Cronbach's α coefficient was used to assess the internal consistency where a value of α above 0.7 is considered as acceptable [30]. Interclass correlation coefficients (ICC) was used for test–retest reliability, in general, values between 0.70/0.75 and 0.9 and greater than 0.90 are indicative of good, and excellent reliability, respectively [31].

##### Content validity

Content validity index (CVI) was used to evaluate the content validity of the scale, including the scale level CVI (S-CVI) and the item level CVI (I-CVI). 9 experts (see above section on expert review process) were asked to score the whole scale and each item on clarity and relevancy on a 4-point Likert scale (0 = undesirable, 1 = somewhat desirable, 2 = desirable, 3 = fully desirable). The CVI was then calculated by the number of the evaluators gave fully desirable and desirable divided by the total number of questions. The cut-off of the S-CVI should be 0.9 or higher, and the I-CVI should be 0.78 or higher [32].

##### Construct validity

Although the QOLCE-16 has been proven to have good construct validity by its original author, EFA and CFA were conducted considering that the translated QOLCE-16-C was applied for the first time in Chinese children with epilepsy.

First, EFA was performed to obtain the factor structure by using principal components analysis with varimax rotation [33]. Before the EFA, Kaiser–Meyer–Olkin’s (KMO) measure and Bartlett’s sphericity were chosen for verification, where a KMO index greater than 0.50 was considered eligible to conduct EFA [34]. Criterions used to determine the number of effective factors were: (1) eigenvalues greater than 1.0, (2) the percentage of total explained variance accounted for, and (3) items with loading greater than 40% in absolute value [33]. Second, CFA was conducted to verify the factor structure determined in the exploratory study. The maximum likelihood estimation method was used to estimate the parameters of the model. Items were first loaded onto 4 factors and then integrated into one factor. The model fit indices are deemed acceptable by χ^2^/df < 3, comparative fit index (CFI) > 0.90, goodness of fit index (GFI) > 0.90 and root-mean-square error of approximation (RMSEA) < 0.08[35]. After that, the convergent validity of each dimension was tested by the values of the average variance extracted (AVE), where the value of AVE higher than 0.5 was considered acceptable, and discriminant validity was determined by comparing the square root value of AVE of each dimension with the correlation coefficient of a particular dimension with those of other dimensions [36].

## Results

### Phase 1: translation and cross-cultural adaptation

#### The forward and backward translation

The results of the three forward translators were able to reached a general consensus for translation, except for the phrase “health and well-being,” and item 1 “Had trouble understanding directions.” Although the words “health” and “well-being” both mean health, but “well-being” also holds a meaning equating to the subjective experience of one’s happiness in life. After further discussion, these words were collectively translated as “health and life quality.” Additionally, there were objections to the translation of “directions,” as it contains both the direction and indication two kinds of meanings. However, since this item belonged to the cognitive dimension, it was more appropriate to translate it as an indication, as cognitive impairment is common in children with epilepsy. During the backward translation, we observed that most of the items and instructions were almost coincided with the original scale, possibly owing to majority being daily expressions with few abstract concepts. The integrated forward and backward version of the QOLCE-16 were delivered for further harmonization and review.

#### Harmonization

The 5-member harmonization team generally agreed with the forward and backward translation, but put some adjustments to better order items in the same dimension. For example, in the original scale, “Had difficulty following complex instructions” came before “Had difficulty following simple instructions.” However, in the Chinese language, it is more common to put the simpler portion first. Therefore, the order of the two items were reversed.

#### Cross-cultural adaption

Five native Chinese parents (one farther, four mothers) whose child had epilepsy were interviewed, ranging in age from 31 to 40 years, with educational levels covering junior high school education to doctoral degrees. Generally, participants reported that all the items and instructions were appropriate without any uncomfortableness. Two parents had differing opinions on one translation respectively: “instructions” was understood as “imperative commands” by one mother, whereas “supervision” was understood as “overprotective” by another mother. Considering that the two mothers were of a low educational level, we believe that it may be an isolated phenomenon. As such, we elected not to revise it and instead the results over to experts for review.

#### Expert review

A total of three rounds of expert reviews were conducted, and all nine experts gave feedback actively. The semantic meaning and content of the scale were examined according to the local Chinese culture. Beyond that, the uncertainties encountered in the translation and cultural adaption process were discussed in detail. The phrase “health and well-being” was eventually translated into “quality of life” in Chinese, as it was consistent with the content measured by the scale. The word “supervision” in the item “Need more supervision than other children in his/her age” was determined to be translated as “caring” for parents can better understand it. In addition, experts determined the expression of the answers to the scale, such as “All of the time” and “Very often” were translate as “always” in Chinese. The Chinese version of QOLCE-16 was obtained in Appendix 1.

### Phase 2: psychometric properties of the QOLCE-16-C

#### Demographic characteristics of the subjects

From the total number of 430 parents, 414 participants from Shanghai Children’s hospital were recruited, excluded those for not filling out sufficient data. Among these 414 epilepsy children, 203 (49%) were boys, with the average age of 9.73 years old and a standard deviation of 3.31 years old, 170 (41.1%) were partial seizures, 173 (41.8%) were generalized seizures, 298 (63.0%) of them took only one or zero antiepileptic drug. Among these 414 respondents, 298 (72.0%) were mothers, 13 (3.1%) were fathers, 103 (24.9%) were grandparents, with age ranged from 27 to 80 (44.2 ± 12.52) years old, 246 (59.42%) of them had a bachelor degrees or above. Of those participants, 203 of them are for the EFA and 211 are for the CFA. The detailed demographic characteristics of the total subjects are presented in Table 1.

**Table 1:** Demographic characteristics of the subjects (n = 414)

Variable	N (%)
*Information about epilepsy children*	
Gender	
Male	203 (49.03%)
Female	211 (50.97%)
Mean age (SD)	9.73 (3.31)
Grade level	
Haven’t started school	4 (1.0%)
Kindergarten	62 (14.98%)
Primary school	235 (56.76%)
Secondary school	91 (21.98%)
High school	14 (3.38%)
Special school	8 (1.93%)
Epilepsy type	
Partial	170 (41.06%)
Generalized	173 (41.79%)
Don’t know	71 (17.15%)
Mean duration of epilepsy (SD)	3.95 (3.19)
Types of AEDs	
0–1	298 (71.98%)
2–3	113 (27.29%)
> 3	3 (0.72%)
*Information about the respondents and families*	
Relationship to child	
Farther	13 (3.14%)
Mother	298 (71.98%)
Grandparents	103 (24.88%)
Mean age (years)	44.24 (12.52)
Education level	
Primary school	4 (0.97%)
Secondary school	56 (13.53%)
High school/Technical school	108 (26.09%)
Undergraduate/Junior College	153 (36.96%)
Master or above	93 (22.46%)
Average monthly household income	
2000–5000	16 (3.86%)
5000–8000	67 (16.18%)
8000–10,000	120 (28.99%)
> 10,000	63 (50.97%)

#### Item analysis

A total of 414 samples were included for item analysis, and there were no missing data for each item. The QOLCE-16-C showed a 0.2% floor effect and 1.0% ceiling effect, which means of no floor or ceiling effect in the Chinese version of QOLCE-16. The item analysis results showed that the CR coefficients of all items were higher than 3 (*P* < 0.001). The scores of all items are significantly related to the total scores (Table 2).

**Table 2:** Floor effect and ceiling effect of each item, item analysis for the QOLCE-16 (n = 414)

Item	Mean ± SD	CR	P	Item-total correlation	*P*
C_1	75.42 ± 24.383	21.095	< 0.001	0.772**	< 0.001
C_2	67.63 ± 28.588	20.769	< 0.001	0.750**	< 0.001
C_3	80.13 ± 21.291	17.563	< 0.001	0.753**	< 0.001
C_4	64.98 ± 28.730	20.324	< 0.001	0.745**	< 0.001
E_1	69.02 ± 20.707	11.327	< 0.001	0.564**	< 0.001
E_2	65.94 ± 22.403	10.374	< 0.001	0.542**	< 0.001
E_3	67.81 ± 21.050	7.904	< 0.001	0.510**	< 0.001
E_4	64.07 ± 22.545	13.436	< 0.001	0.639**	< 0.001
S_1	79.05 ± 25.867	17.048	< 0.001	0.639**	< 0.001
S_2	71.62 ± 22.897	19.312	< 0.001	0.791**	< 0.001
S_3	72.40 ± 24.186	18.719	< 0.001	0.775**	< 0.001
S_4	73.55 ± 23.231	21.491	< 0.001	0.797**	< 0.001
P_1	67.75 ± 21.910	18.551	< 0.001	0.749**	< 0.001
P_2	63.83 ± 23.481	19.205	< 0.001	0.752**	< 0.001
P_3	70.59 ± 22.687	15.229	< 0.001	0.700**	< 0.001
P_4	61.65 ± 24.264	11.948	< 0.001	0.530**	< 0.001

**Correlation is significant at 0.01 level (2-tailed)

#### Reliability

The results shown that the Cronbach’s α coefficients for the total and four dimensions of the QOLCE-16-C were 0.938, 0.945, 0.814, 0.922, and 0.891 respectively. There were 32 people completed the retest survey. The ICC for the total and each dimension were 0.724, 0.716, 0.615, 0.650, and 0.672 respectively (Table 3).

**Table 3:** The reliability of the QOLCE-16

	Cronbach’s α (n = 414)		Test–retest reliability (n = 32)
	Canadian version	Chinese version	
Cognitive	0.92	0.945	0.716
Emotion	0.75	0.814	0.615
Social	0.85	0.922	0.650
Physical	0.80	0.891	0.672
Total	0.90	0.938	0.724

#### Validity

##### Content validity

The positive coefficients of the 9 experts were 100%. The S-CVI and the I-CVI was 1, except for item 5, which was 0.89, indicating that the importance of all items in the QOLCE-16-C has reached an expert consensus.

##### Construct validity

The exploratory factor analysis shown that the KMO value was 0.928, Bartlett’s test χ^2^ = 5514.698, *P* < 0.01, which was suitable for exploratory factor analysis. Principal component analysis method was used to extract 4 common factors. The characteristic values after the axis were 3.573, 3.145, 3.131, 2.660, all greater than 1. Its cumulative variance contribution rate was 78.183%, which meets the requirement that the cumulative variance contribution rate to be at least 40% [33]. The factor loading of each item was between 0.621 and 0.877. Table 4 shows more details.

**Table 4:** Factor loadings of Chinese version of QOLCE-16 (n = 203)

Items	Factor 1	Factor 2	Factor 3	Factor 4
*Cognitive*				
C_2	**0.877**	0.239	0.245	0.120
C_1	**0.868**	0.246	0.237	0.199
C_4	**0.847**	0.231	0.199	0.173
C_3	**0.799**	0.255	0.292	0.202
*Social*				
S_4	0.269	**0.807**	0.324	0.192
S_2	0.235	**0.794**	0.312	0.250
S_3	0.274	**0.767**	0.313	0.199
S_1	0.333	**0.728**	0.213	0.227
*Physical*				
P_1	0.296	0.244	**0.839**	0.175
P_3	0.287	0.241	**0.797**	0.130
P_2	0.337	0.243	**0.757**	0.206
P_4	0.099	0.287	**0.720**	0.072
*Emotional*				
E_2	0.213	0.041	0.107	**0.838**
E_1	0.104	0.169	0.196	**0.787**
E_3	0.161	0.221	0.009	**0.737**
E_4	0.082	0.413	0.255	**0.621**
Characteristic values	3.573	3.145	3.131	2.660
Variance contribution (%)	22.332	19.659	19.566	16.626
Cumulative (%)	22.332	41.991	61.557	78.183

The bold parts indicate that they belong to the same factor

The CFA was performed to test the fitness of the total scale. The construct of the total scale was acceptable for χ^2^/df = 1.698, GFI = 0.913, CFI = 0.974, RMSEA = 0.058 (Table 5). The value of AVE of each dimension, the square root of every AVE value, and correlation coefficients between dimensions are shown in the Table 6. It was revealed that the square root of AVE was significantly higher than the dimensions of other correlated coefficients, thus suggesting acceptable discrimination validity.

**Table 5:** Fitness of the QOLCE-16 (n = 211)

χ^2^	df	*P*	χ^2^/df	CFI	GFI	RMSEA
166.378	98	0.000	1.698	0.974	0.913	0.058

**Table 6:** Discriminative validity analysis (n = 211)

	AVE	Cognitive	Emotional	Social	Physical
Cognitive	0.825	**0.908**			
Emotional	0.458	0.503	**0.677**		
Social	0.704	0.632	0.654	**0.839**	
Physical	0.714	0.74	0.552	0.745	**0.845**

The bold parts are the square root of AVE

## Discussion

To the best of our knowledge, this study presents the first attempt to translate the QOLCE-16 into Chinese, tests its validity and reliability among Chinese children with epilepsy.

The steps of cross-cultural translation are rigorous and have firm basis. The 10-step translation method of the guidelines for the Principles of Good Practices for translation and cultural adaptation was used to translate QOLCE-16, which provides effective guidance for cross-cultural translation. Few items were adjusted during the process based on the results of parents' cognitive interviews and the opinions given by expert consultants, such as translating “supervision” to “caring” for better understanding. In addition, the order in which items were placed had been adjusted according to the cognitive habits of Chinese. Consequently, the translated scale can convey the meaning smoothly in the semantics and is also acceptable in the culture.

The QOLCE-16-C had no floor or ceiling effects, with items being well identified and strongly correlated to the total scale. The results of item analysis illustrated that the CR values of each item were greater than 3, which meets the requirement of CR values being greater than 3 and statistically significant [29]. Moreover, there was a strong correlation between the scores of each item and the total scores, indicating that all items were valid.

Internal consistency and test–retest reliability of the QOLCE-16-C are acceptable. The Cronbach’s α coefficients for each dimension was higher than 0.7, and the cognitive and social dimensions were higher, 0.945 and 0.922 respectively, which was consistent with the results of Goodwin (value of 0.92 and 0.85) [16]. The test–retest reliability was completed among 32 parents, and the results shown that the ICC between the two tests ranged between 0.615 and 0.716. The lower correlation may be related to the length of the interval in our study (2–3 weeks). In addition, the way of those retest data been collected may also have contributed to the low correlation, as more than half of the retest data was collected through WeChat electronic questionnaires.

Content validity and construct validity are reasonable. The S-CVI in QOLCE-16-C reached 1, and the I-CVI was 0.89–1, indicating good expert content validity. In structural validity, EFA produced four common factors, which were highly consistent with the results of the original scale. The results of CFA were acceptable for χ^2^/df = 1.698, GFI = 0.913, CFI = 0.974, RMSEA = 0.058. In addition, the QOLCE-16-C also showed good discriminative validity. In future studies, the measurement equivalence of the QOLCE-16-C should be further verified by variety of groups, such as differing in age, gender or time [8].

Finally, we have to reiterate the purpose of the development of QOLCE-16 proposed by the original author Goodwin: to simplify QOLCE-55 and to provide an easy-to-use measurement tool for clinic. The QOLCE-55 is a simplified version of the original 76-item scale and provides an overall assessment of the HRQOL of 4–18 years old children with epilepsy reported by parents, which covering the four genres of HRQOL: cognitive (22 items), emotional (17 items), social (7 items), and physical (9 items) [14]. The scoring method of QOLCE-55 is consistent with QOLCE-16, and it also has excellent internal consistency reliability (α = 0.96). Therefore, QOLCE-55 is a preferred measure in cases where specific domain information or a more comprehensive HRQOL assessment is required. In contrast, QOLCE-16 is more suitable for focusing on aggregate summary scores [16].

There are several limitations of this study need to take further consideration. (1) Convenience sampling survey conducted in only one children's hospital, which may have some impacts on the scale's generalizability. (2) The sample size of the 5 cognitive interviewees we included were relatively small, compared to the recommended acceptable range in the literature being 10–15 subjects [37]. This may result in an unreasonable translation and expression of items. (3) Convergent and divergent validity were performed on 4 dimensions, but did not on the total QOLCE-16-C, which would have required a comparison of the QOLCE-16-C scores with other validated measures. Future studies need to be carried out in different groups (e.g., age, sex, and time) in order to further confirm its applicability in Chinese children with epilepsy.

## Conclusions

In conclusion, this study conducted a translation of QOLCE-16 in China, along with its reliability and validity tests in Chinese children with epilepsy. The contents and items of the QOLCE-16-C are simple and easy to understand. This simplicity was largely due to the small items’ effectiveness in reducing time and effort in two key areas-answering and reporting. After verification, the QOLCE-16-C has been proved to have good reliability and validity, and is suitable for the evaluation of health-related quality of life of children with epilepsy in China. Future studies need to be carried out in different groups to make further confirmation on its applicability in Chinese children with epilepsy.

## Data Availability

The datasets generated and/or analyzed during the current study are not publicly available to preserve the privacy of the participants but are available from the corresponding author upon reasonable request.

## Abbreviations

HRQOL: Health related quality of life
QOLIE-AD-48: Quality of Life in Epilepsy for Adolescents
QOLCE-55: Quality of Life in Childhood Epilepsy Questionnaire
CHEQOL-25: Quality of Life Measure for Children with Epilepsy
QOLCE-16: Quality of Life Childhood Epilepsy Questionnaire
QOLCE-16-C: Chinese version of the Quality of Life in Childhood Epilepsy Questionnaire
ISPOR: International Society for Pharmacoeconomics and Outcomes Research
PROMIS: Patient-reported outcome measurement information system
WHOQOL: World Health Organization Quality of Life
Neuro-QoL: Neuro-quality of life
CR: Critical ratio
ICC: Interclass correlation coefficients
CVI: Content validity index
S-CVI: Scale level of content validity index
I-CVI: Item level of content validity index
EFA: Exploratory factor analysis
CFA: Confirmatory factor analysis
KMO: Kaiser–Meyer–Olkin
CFI: Comparative fit index
GFI: Goodness of fit index
RMSEA: Root-mean-square error of approximation
AVE: Average variance extracted
